# Research on a high-efficiency composite heald frame with carbon fiber reinforcement

**DOI:** 10.1371/journal.pone.0354527

**Published:** 2026-08-28

**Authors:** Qiu Haifei, Zhang Quan, Nie Zhike, Deng Ruoqing, Li Guozheng

**Affiliations:** 1 School of Mechanical Engineering, Xijing University, Xi’an, China; 2 Engineering Research Center of Hydrogen Energy Equipment and Safety Detection, Universities of Shaanxi Province, Xijing University, Xi’an, China; King Mongkut’s University of Technology North Bangkok, THAILAND

## Abstract

To satisfy the high-speed production demand of modern looms, a sandwich-structured delamination composite heald frame was fabricated using carbon fiber, epoxy resin, honeycomb core, wood core plates and damping sealing strips. With the interleaved laying scheme of unidirectional carbon fiber prepreg and circular winding technology adopted, a finite element model of the composite inner beam was constructed on the WorkBench/ACP platform. Modal analysis revealed that symmetric layup delivers higher low-order natural frequencies (1st–5th orders) and better dynamic performance than asymmetric layup. On this basis, a multivariate dynamic optimization model with variables of layup angle *α*, *θ* and layup thickness *t* was established, and an APDL-based parametric finite element model was built for asymmetric carbon fiber layup. The sub-problem algorithm was applied to optimize the carbon fiber layup parameters, raising the 1st–6th order natural frequencies of the composite beam by 16.6–335 Hz and validating the reliability of the proposed optimization model. Vibration, noise and cam wear tests further verified that the carbon fiber-reinforced polymer (CFRP) composite heald frame outperforms the conventional YG4 steel heald frame in vibration and noise reduction, as well as loom energy conservation. This research provides a theoretical and technical reference for the structural design and manufacturing of novel composite heald frames.

## 1. Introduction

During the weaving process, the heald frame operates in a state of high-speed reciprocating motion for an extended period. The vibration, noise, and fatigue damage induced by this motion not only cause fluctuations in yarn tension and compromise fabric quality but also tend to accelerate component wear and result in technical failures of the shedding mechanism [1]. In recent years, with the continuous advancement of textile automation, the speed of modern shuttleless looms has reached up to 1800 r/min (e.g., the ZAX air-jet loom manufactured by TSUDA KOMA Corporation) [2]. Against this backdrop, traditional heald frames (including wooden, iron, and aluminum alloy types) can no longer satisfy the production demands of high-speed looms.

To address the speed matching issue between the heald frame and high-speed looms, it is essential to overcome the performance limitations of traditional heald frames through innovations in new materials and processes. Scholars, institutions, and universities in the industry have conducted extensive exploratory work in the research and development of new heald frames. Over the long term, they have achieved certain research progress, which are detailed as follows: Lee D G et al. from South Korea have designed and developed a composite material heald frame suitable for high-speed air-jet looms. By improving the weight and structural rigidity of the heald frame, they have effectively promoted the increase in the running speed of the air-jet looms [3]. Inokuchi Hirokazu and Fujii Mikya of YKK Corporation, Japan, found through experimental research that under the same loom speed, the vibration noise and component wear caused by carbon fiber composite heald frames are significantly lower than those caused by steel heald frames [4]. Huo F L from China prepared hybrid sandwich structure composite heald frame test specimens using mold pressing technology, and studied the influence of different layup schemes and reinforcing materials on the vibration performance of the heald frame through finite element analysis, vibration tests, and bending tests [5]. Han B B et al. from China have conducted dynamic design and analysis of aluminum alloy, carbon aluminum composite, and carbon fiber composite heald frames, obtaining the dynamic characteristics of three different materials and structural types of heald frames, thereby providing a strong basis for the dynamic design of new heald frames [6]. However, the aforementioned studies did not address some key technologies related to sandwich composite frames, such as fiber layer winding, parametric modeling of fiber layers, dynamic optimization, and novel manufacturing processes, which are precisely the issues this paper focuses on and aims to resolve.

Currently, there are already some composite heald frames available on the market. For example, the Swiss company Grob has developed a hybrid structural composite heald frame (WXS), which is made of high modulus carbon fiber composite materials and steel. Experiments have shown that this new composite heald frame has the characteristics of being lightweight, high-strength, and low-vibration. The American company Steel Heddle has also successfully developed a new type of carbon fiber heald frame and conducted performance tests at a speed of 900r/min, which meet the performance requirements of high-speed jet looms very well. Tailun Company in Shenzhen, China has developed a new type of heald frame using carbon fiber and basalt fiber reinforced epoxy resin. It not only features low vibration, low noise, and low energy consumption, but also has a service life that is 3 ~ 4 times longer than that of aluminum alloy heald frame.

However, due to factors such as process complexity, raw material costs, and loom speed, most textile enterprises still adopt third-generation aluminum alloy heald frames. The theoretical research, design methods, and preparation processes of composite heald frames are not yet mature; in particular, the application technology of carbon fiber-reinforced resin materials in heald frame manufacturing urgently needs breakthrough [7]. There is still a long way to go for the promotion and application of new composite heald frames.

In this paper, a composite crossbeam with sandwich delamination was applied to the technological upgrading of the carbon fiber reinforced polymer (CFRP) heald frame. This study contributes to the lightweight design and cost control of composite heald frames, and provides important insights for the development of new-generation heald frames.

## 2. Method and principle

### 2.1. Weaving principle

**Fig 1:**
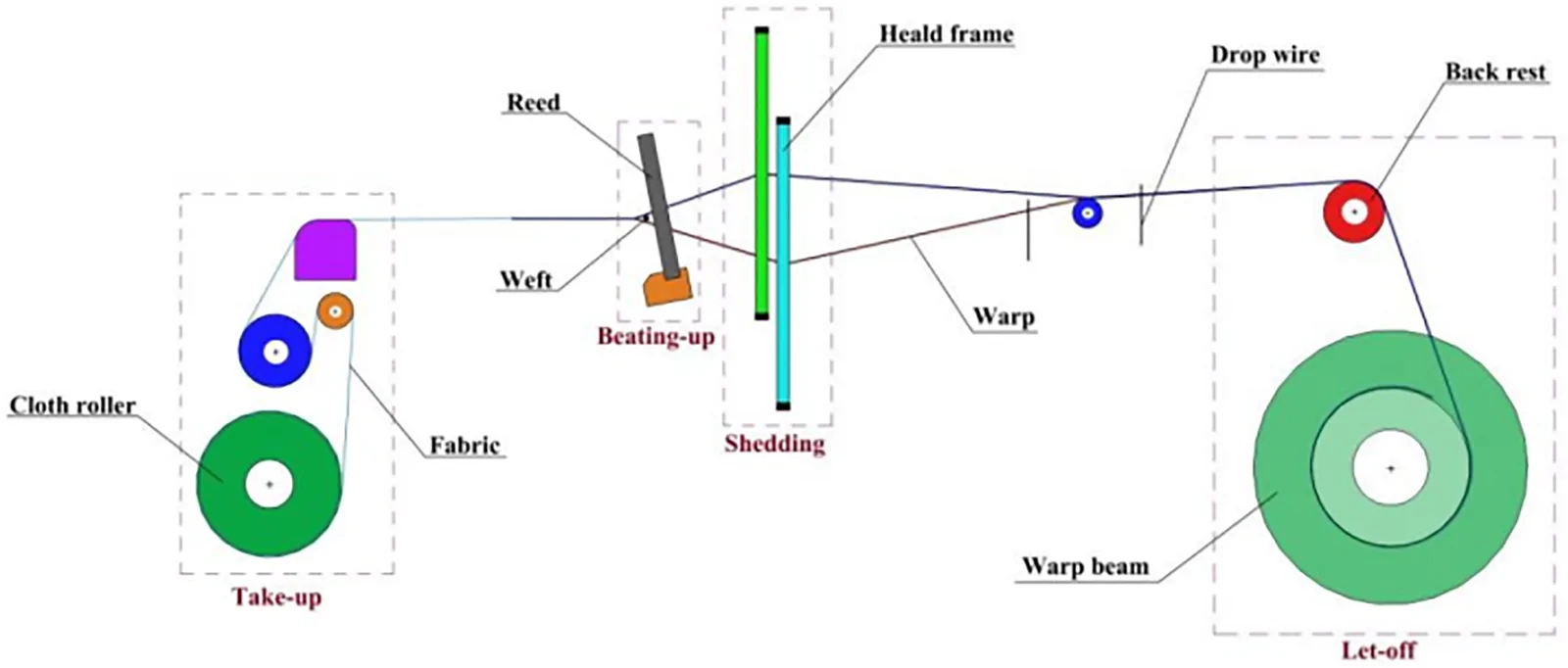
Weaving principle diagram.

As a core moving component of the shedding system, the heald frame significantly impacts the weaving efficiency and fabric quality. When the loom operates at high speed, the heald frame undergoes reciprocating motion along the vertical direction driven by cams or linkages [8], as illustrated in Fig 1, where *v*_t_ represents the instantaneous upward velocity of the heald frame, and *v*_d_ represents its instantaneous downward velocity.

With the coordinated movement of the let-off and take-up system, the warp yarns quickly separate and form a shed under the pulling action of the heald frame. Simultaneously, the weft insertion system rapidly passes the weft yarn through the shed with a certain width. Subsequently, the reed swings back and forth to beat the weft yarns one by one, causing them to interlace with the warp yarns and form fabric. Finally, the fabric is continuously wound onto the cloth roller.

### 2.2. Heald frame structure

**Fig 2:**
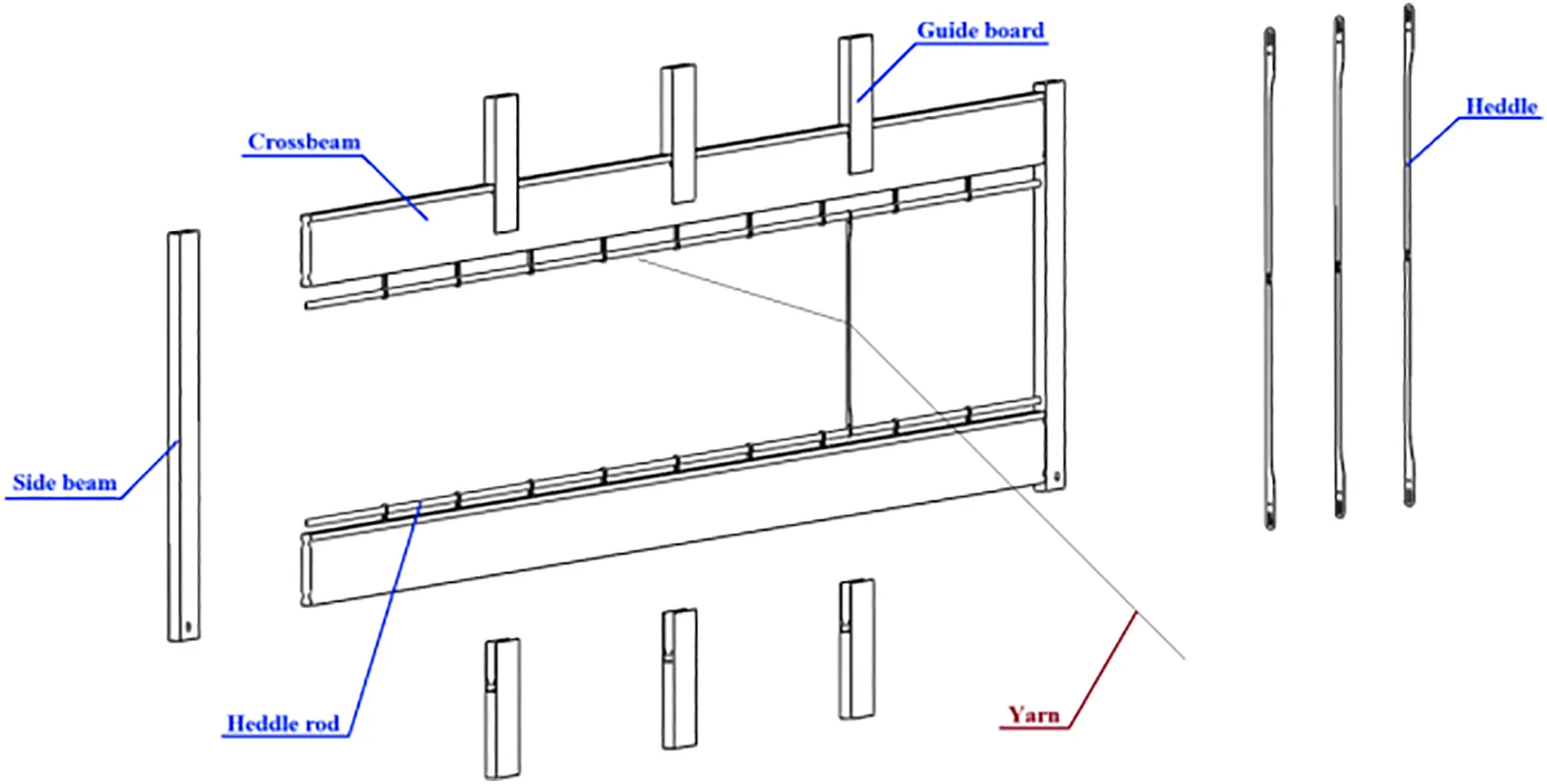
Composition of the heald frame.

The heald frame is a typical planar structure, mainly consisting of crossbeams, guide boards, side beams, heddle rods, and heddles, as illustrated in Fig 2. As the core structural component of the heald frame, the crossbeam provides a mounting base for other components, which are assembled on it in accordance with their functional requirements [9]. The length of the crossbeam is mainly determined by the width of the loom. Currently, the width of shuttleless looms ranges from 220 cm to 460 cm, while advanced wide-width looms can even exceed 540 cm in width.

An ideal heald frame should possess characteristics such as light weight, low vibration and noise, high rigidity, and high fatigue strength. Previous research and production practices have shown that the vibration and noise of the loom system mainly originate from the heald frame, and the dynamic characteristics of the heald frame are mainly manifested as bending and torsional vibration of the crossbeam [10]. Therefore, the dynamic behavior of the crossbeam on the heald frame is crucial for reducing vibration and noise, as well as ensuring stable high-speed operation of the looms.

### 2.3. Composite crossbeam

Carbon fiber reinforced polymer (CFRP) possesses excellent mechanical and physical properties, such as low weight, high specific modulus, high specific strength and outstanding fatigue resistance [11]. Table 1 lists the mechanical parameters of different carbon fiber grades (UHM, UHT, HM, HT, IM), and all carbon content and mechanical indexes provided in this table are sourced from the composite material monograph [12]. CFRP delivers a specific stiffness 3.10 times higher than steel and 3.18 times higher than aluminum alloy, with intrinsic damping 10–50 times larger than metallic materials. Under equivalent stiffness constraints, CFRP greatly reduces the mass of crossbeams to match the high reciprocating speed of heald frames. Moreover, its superior fatigue performance delays structural failure and extends the overall service life of looms under long-duration weaving operation.

**Table 1:** Mechanical properties of carbon fiber.

Mechanical property	UHM	UHT	HM	HT	IM
Strength of extension/[GPa]	>400	200 ~ 350	300 ~ 400	200 ~ 250	180 ~ 200
Modulus of extension/[GPa]	>1.7	>2.76	>1.7	2.0 ~ 2.75	2.7 ~ 3.0
Carbon content/%	99.8	96.5	99.0	96.5	99.0

**Fig 3:**
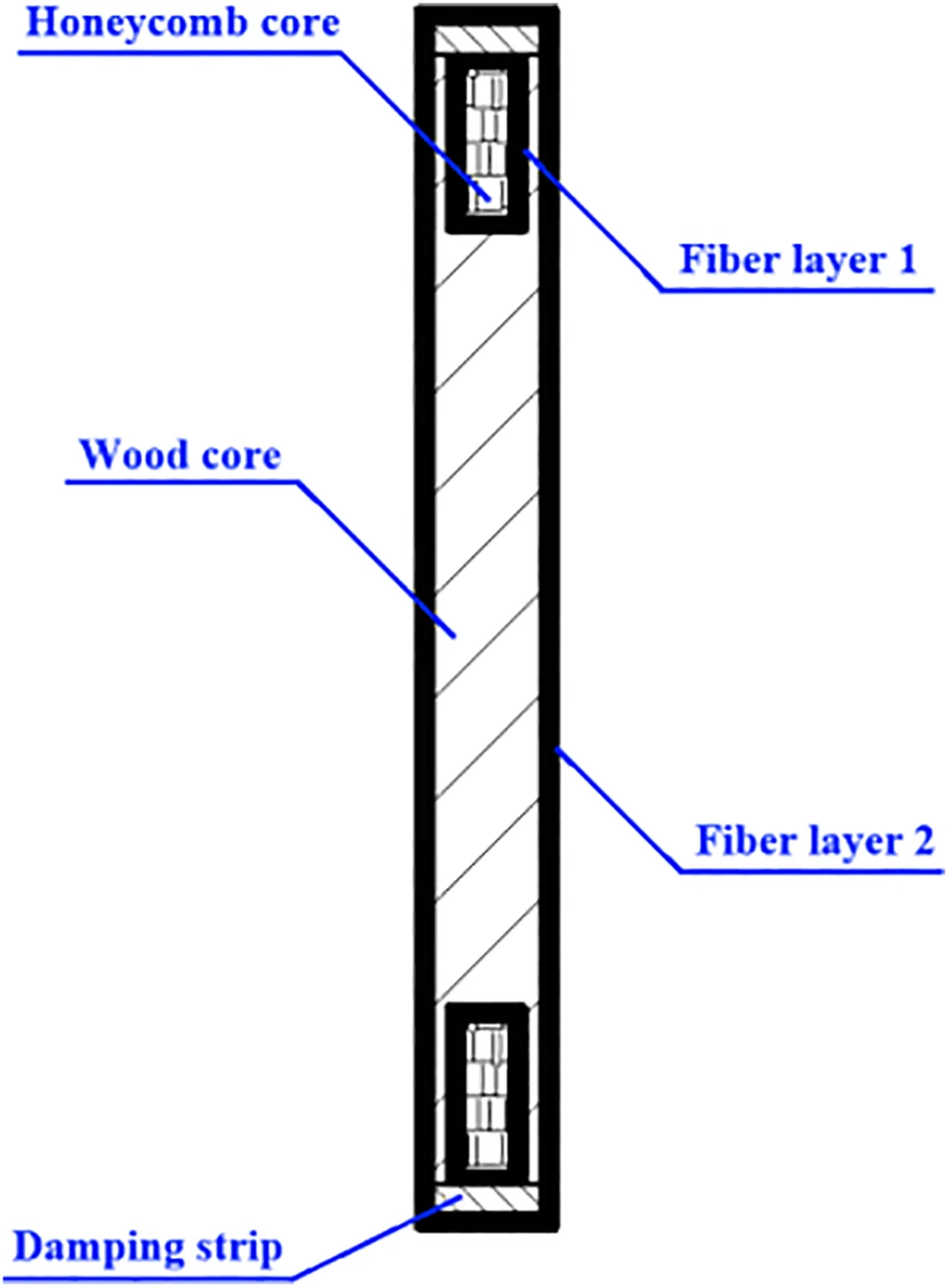
Cross-section of the composite crossbeam.

**Fig 4:**
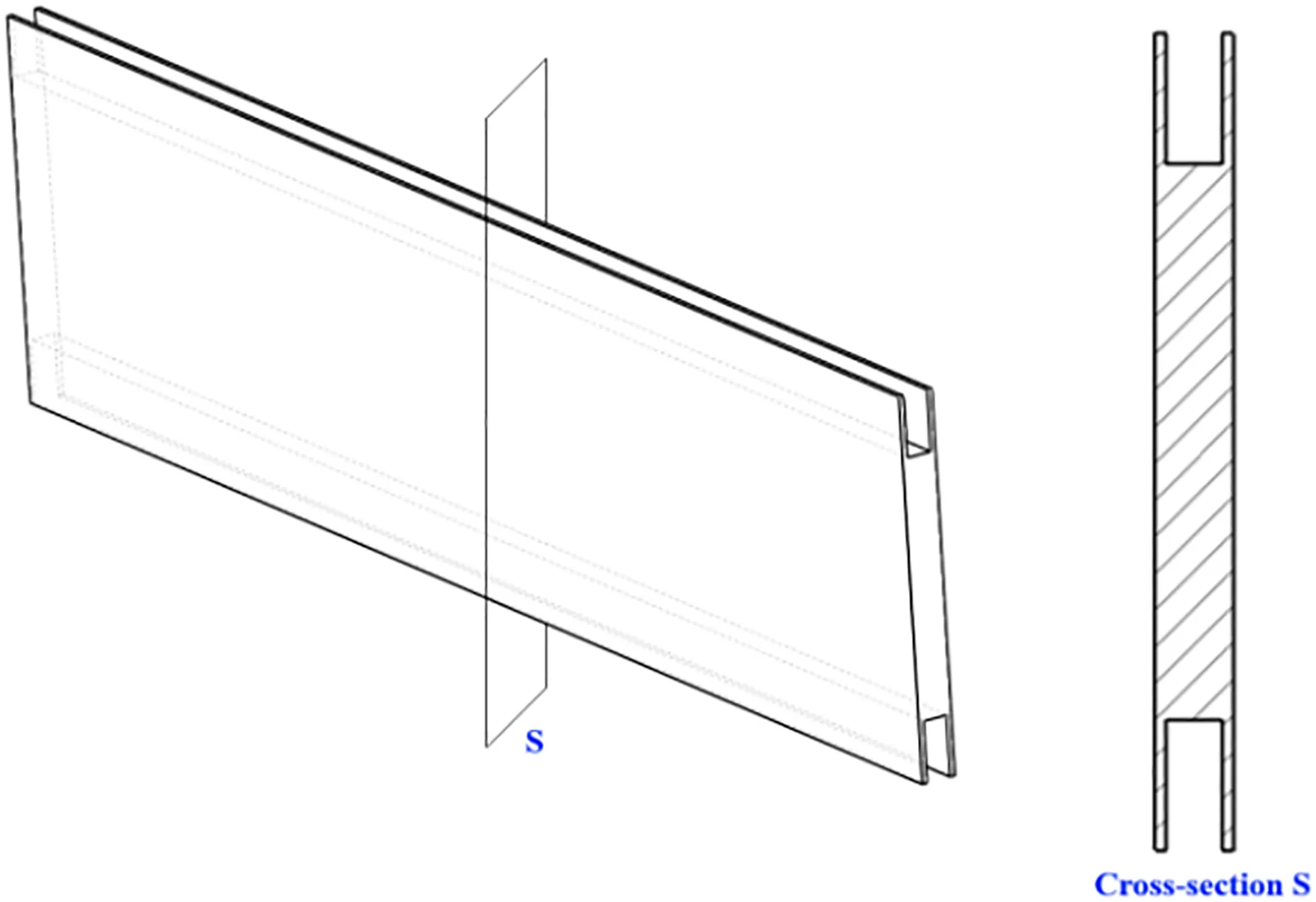
Wood core panel.

In this paper, a composite crossbeam was designed for a new heald frame, as shown in Fig 3. The crossbeam is made by mixing core materials with carbon fiber prepreg, and its main structure is a wooden panel with U-shaped grooves at both ends (see Fig 4). Inside the U-shaped groove, a composite inner beam is bonded to enhance the strength and stability of the heald frame. Different from beam structures made of a single material, this inner beam is composed of honeycomb core and carbon fiber prepreg (Fiber layer 1) wound in layers on its outer surface.

To further improve the vibration damping performance of the crossbeam, a damping strip with both vibration reduction and sealing functions is bonded to the top of the U-shaped groove. Additionally, several layers of carbon fiber prepreg (Fiber layer 2) of a certain thickness is wrapped around both sides of the wood core and the outer surface of the damping strip. The dual-core (wood core and honeycomb core) layered structure in this paper significantly reduces the amount of carbon fiber used in the composite crossbeam. This not only helps achieve lightweighting of the crossbeam but also effectively lowers the R&D cost of the composite heald frame.

### 2.4. Prepreg layup process

#### 2.4.1. Laminated structure.

**Fig 5:**
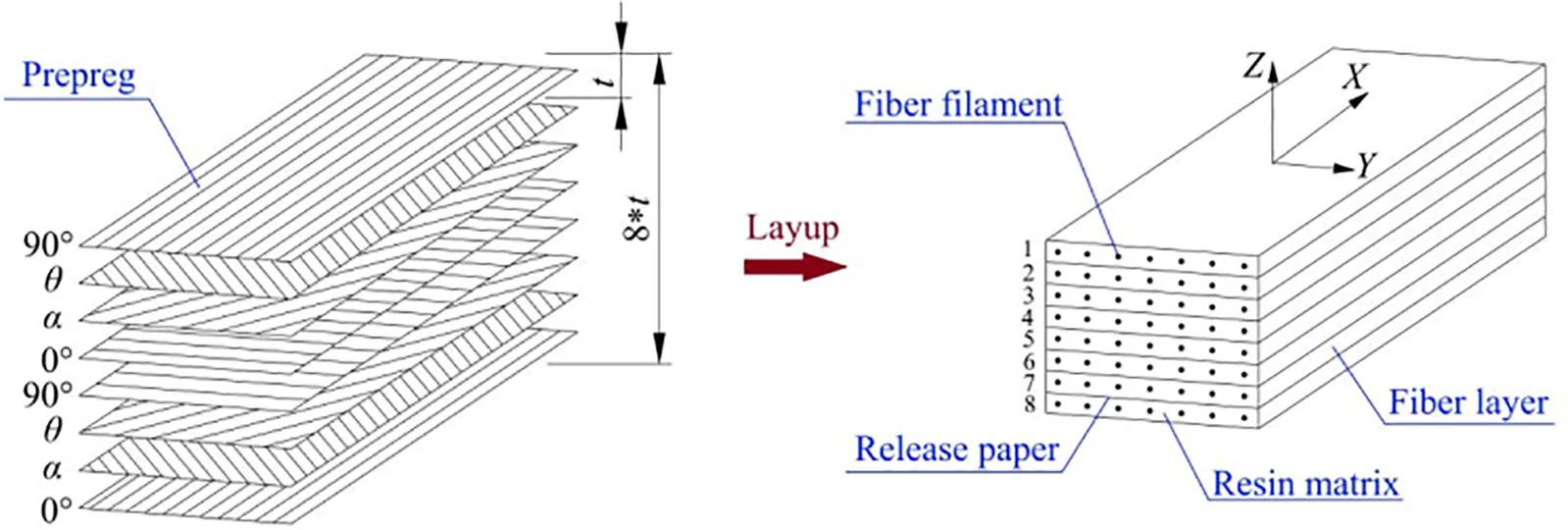
Carbon fiber prepreg layup.

By stacking several layers of prepreg with different fiber orientations in a specific order and then injecting uncured epoxy resin or other thermosetting polymer matrix materials, the most basic fiber-reinforced composite material can be produced [13]. This paper prepares composite crossbeams by laminated winding of carbon fiber prepreg, which consists of release paper, carbon fiber filament, and a resin matrix material (epoxy resin), as illustrated in Fig 5.

A complete layup unit consists of 8 layers of carbon fiber prepreg, with the layup angle specified as [0/*α*/*θ*/90/0/*α*/*θ*/90], where *t* represents the thickness of a single layer of carbon fiber prepreg. *α* and *θ* denote the fiber laying angles in the 2nd/3rd and 6th/7th prepreg layers, respectively, with their values ranging from 25° to 65°.

The manufacturing process of the inner beam is as follows: first, carbon fiber prepreg coated with resin adhesive is laid in parallel and wrapped around the outer surface of the honeycomb core. Subsequently, a roller is used to smooth the fabric surface and remove air bubbles until the prepreg is tightly bonded to the outer surface of the honeycomb core [14]. Using the same process, the remaining prepreg layers are sequentially laid and wrapped until the required thickness of fiber layer 1 for the inner beam is achieved (i.e., x*t, where x is an integer multiple of 8). Finally, the inner beam component is placed into a mold coated with release agent, locked and clamped, and then heated to cure and pressurized to shape in an oven (within a temperature range of 80°C to 130°C). After the mold and carbon fiber layers are fully cured and shaped, they are removed from the oven, the mold is opened, and the inner beam component is allowed to cool naturally in the air. The layup process and preparation method of fiber layer 2 are the same as those of fiber layer 1, and thus will not be elaborated further.

#### 2.4.2. Laying sequence and direction.

**Fig 6:**
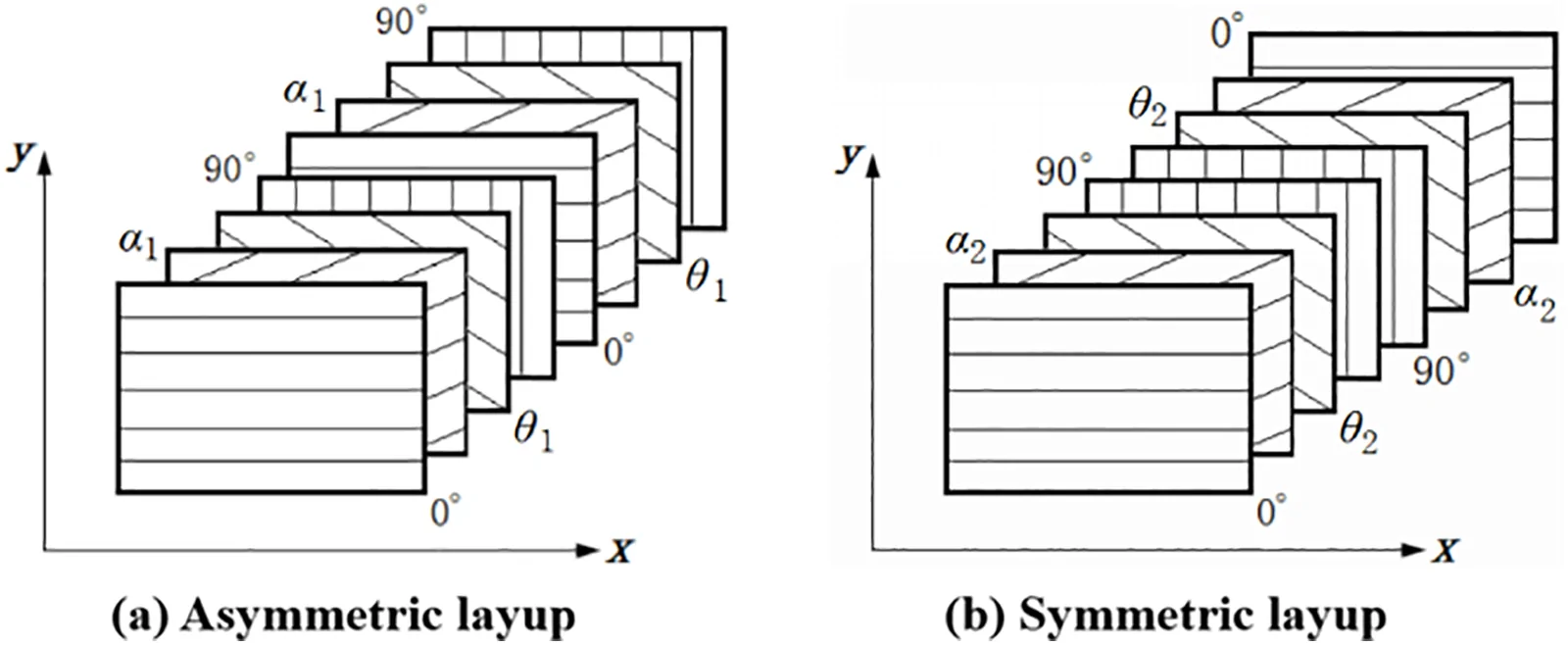
Prepreg layup sequence and direction. (a) Asymmetric layup (b) Symmetric layup.

To enhance the overall strength and stiffness of the crossbeam on the heald frame, an asymmetric layup (fiber layer 1) is adopted for the winding of carbon fiber prepreg on the outer surface of the honeycomb core, as illustrated in Fig 6(a). A complete carbon fiber laminated structure consists of 8 layers of prepreg, with the laying direction referenced by the angle between the fiber threads and the *X*-axis, denoted as [0/*α*_1_/*θ*_1_/90/0/*α*_1_/*θ*_1_/90]. Here, the thickness of each carbon fiber prepreg layer is t1, and x*t_1_ is approximately one-quarter of the width of the U-shaped groove. To distinguish between the 0° and 90° fiber layers, the value ranges of *α*_1_ and *θ*_1_ are set to [25°, 65°].

To eliminate the bending coupling stress caused by the transverse vibration of the heald frame, carbon fiber prepreg with a symmetric layup (fiber layer 2) is applied to the outer surface of the crossbeam, as shown in Fig 6(b). The layup angle is referenced to the angle between the fiber threads and the *X*-axis, denoted as [0/*α*_2_/*θ*_2_/90/90/*θ*_2_/*α*_2_/0], where the thickness of each prepreg layer is denoted as *t*_2_, and x*t_2_ is determined by the width of the guide rail groove of the heald frame. The value ranges of *α*_2_ and *θ*_2_ are also set to [25°, 65°].

The main difference between carbon fiber layer 1 and carbon fiber layer 2 is their layup sequence and angles. Layer 1 uses an asymmetric fiber layup:[0/*α*_1_/*θ*_1_/90/0/*α*_1_/*θ*_1_/90], while layer 2 employs a symmetric fiber layup:[0/*α*_2_/ *θ*_2_/90/90/*θ*_2_/*α*_2_/0]. Compared to single-layer laminated structures and layup processes, this dual-core crossbeam that simultaneously combines asymmetric and symmetric layups can enable the heald frame to have more excellent comprehensive working performance [15].

## 3. Discussion and analysis

### 3.1. Dynamic characteristics

#### 3.1.1. Finite element modeling.

The finite element model of the inner beam component was set up, and a preliminary numerical investigation of its dynamic characteristics was conducted using ANSYS/WorkBench software. The composite inner beam was designed and fabricated with “Epoxy Carbon UD 230 GPa Prepreg and honeycomb core” (the material available in the Composite Materials module of WorkBench/ACP) via prepreg interleaved layup and bonding processes. The material performance parameters are presented in Table 2.

**Table 2:** Material property parameter.

Material	Density/ kg·m^-3^	Young’s modulus/GPa			Shear modulus/GPa			Poisson’s ratio		
		*E*_*x*_	*E*_*y*_	*E*_*z*_	*G*_*xy*_	*G*_*xz*_	G_*yz*_	*γ*_*xy*_	*γ*_*xz*_	*γ*_*yz*_
Prepreg	1490	121	8.6	8.6	4.7	4.7	3.1	0.27	0.27	0.4
Honeycomb	80	0.001	0.001	0.255	1 × 10^−9^	0.07	0.037	0.49	0.001	0.001

**Fig 7:**
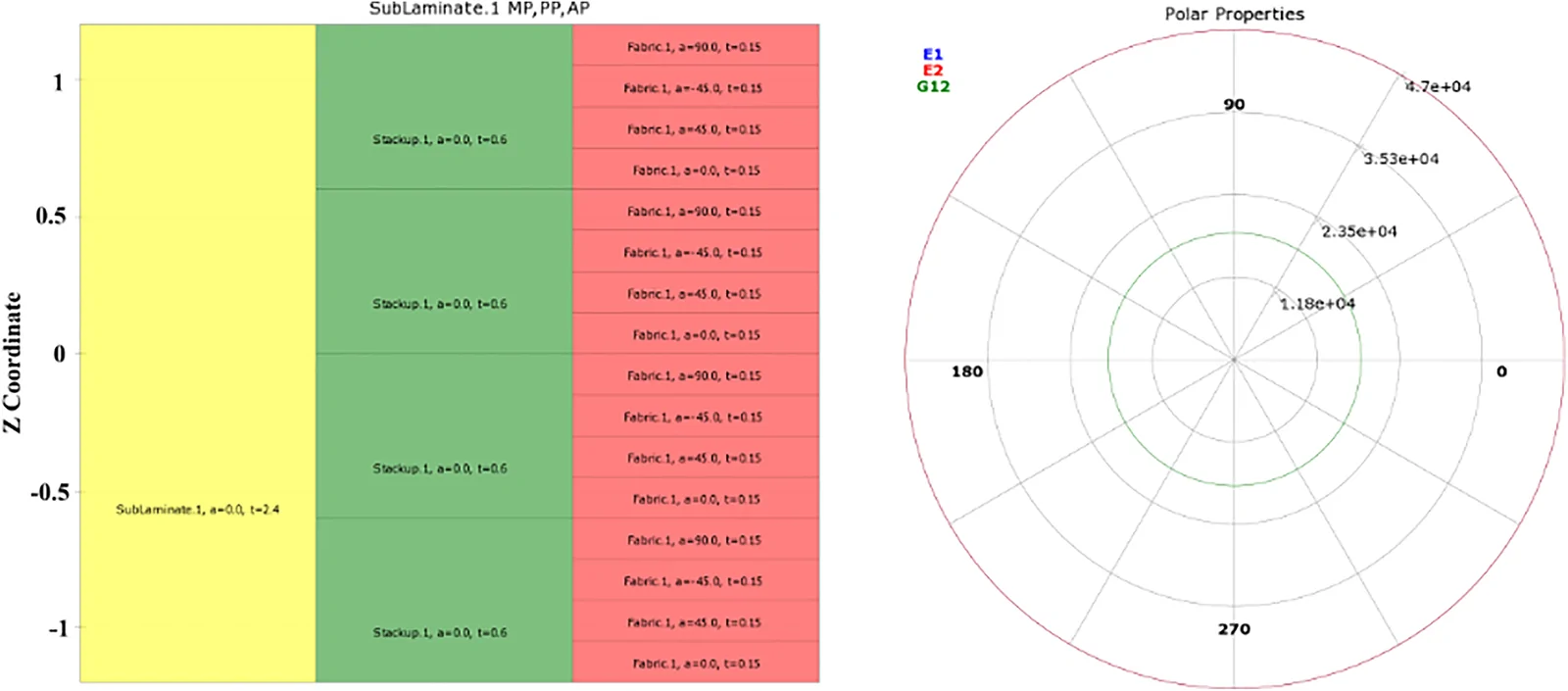
Fiber layup scheme of laminate.

Carbon fiber layer 1, which is required for preparing the composite inner beam using asymmetric layup (see Fig 6a), has a laminated structure consisting of four Stackup basic units, as illustrated in Fig 7. Each Stackup basic unit is formed by bonding four layers of carbon fiber prepreg, with a layup angle of [0/45/-45/90] and a thickness (*t*_1_) of 0.15 mm.

**Fig 8:**
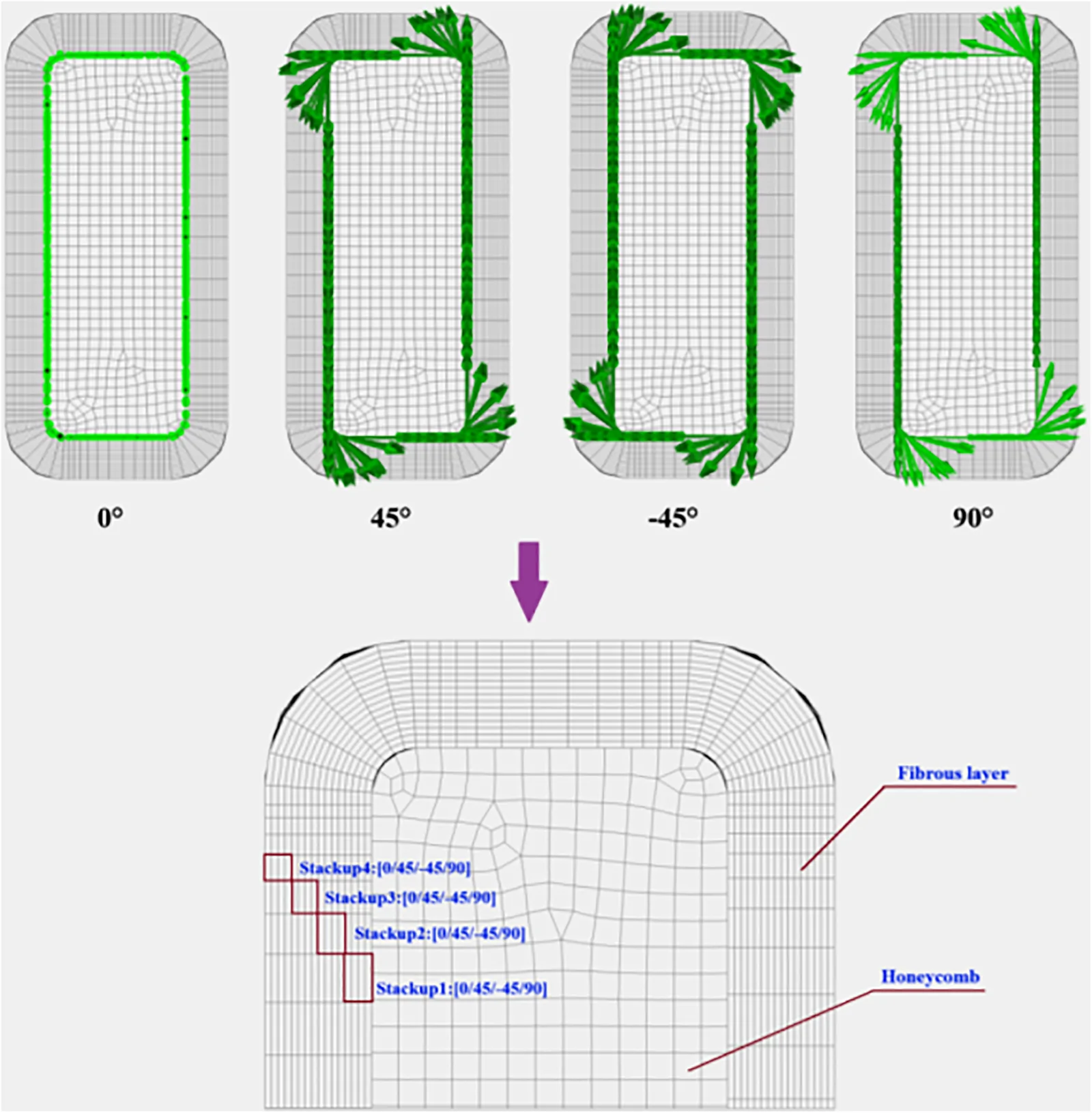
Cross-section of the mesh model.

To save machine time and computing power, the length of the inner beam was set to 750 mm, which is half the width of a 150 cm loom. Carbon fiber prepreg was sequentially stacked on the outer surface of the honeycomb core, with each prepreg layer meshed, as illustrated in Fig 8. Carbon fiber layer 1 comprises 16 layers of carbon fiber prepreg, each containing unidirectionally laid fiber bundles. The green arrows in the figure indicate the fiber laying direction of each layer.

**Fig 9:**
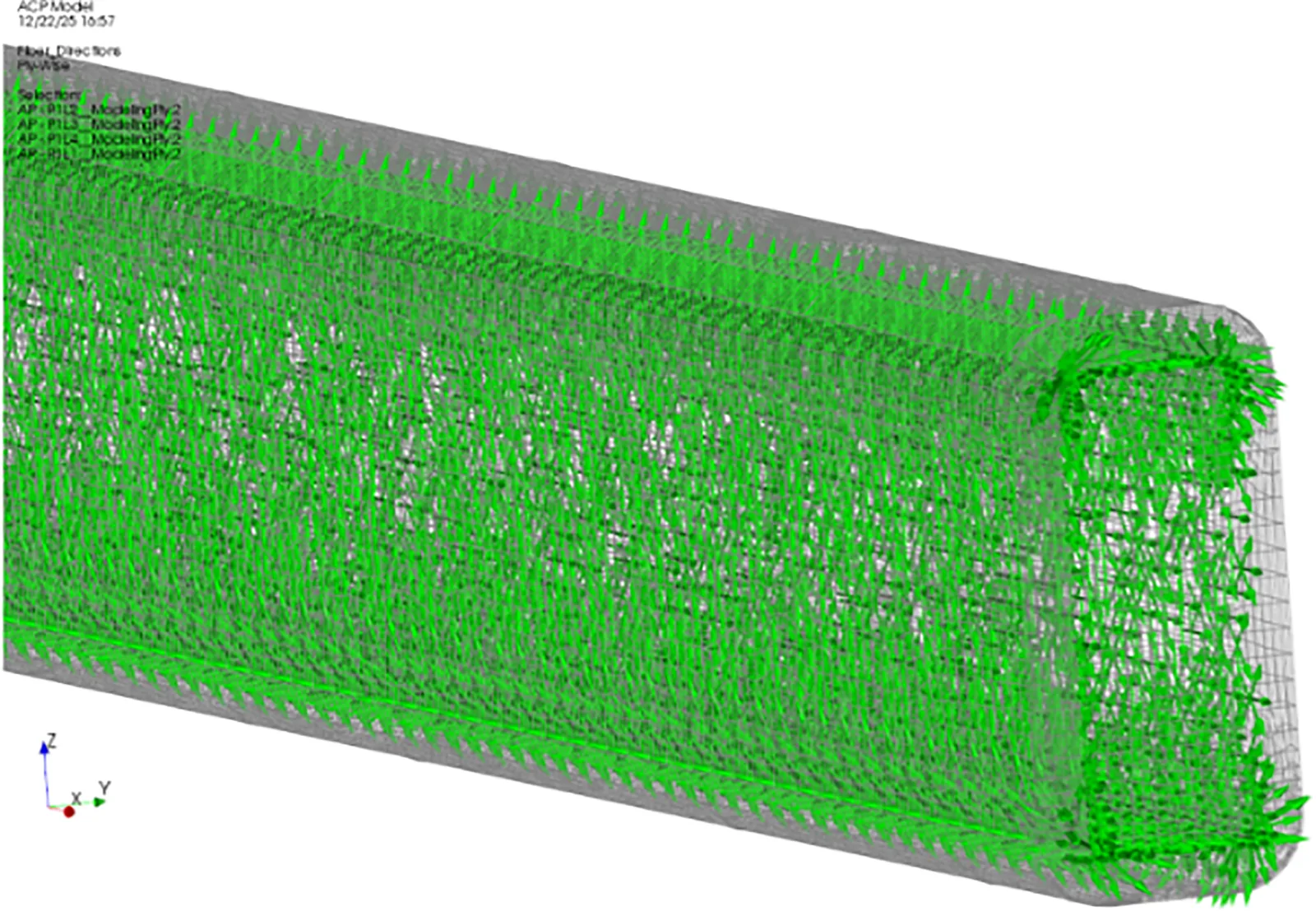
Finite element model of the inner beam.

The thickness of carbon fiber layer 1 is 2.4 mm, with a layup angle of [0/45/-45/90/0/45/-45/90]. As illustrated in Fig 9, the mesh generation results in a total of 1,282,372 elements and 1,351,635 nodes. Among these, carbon fiber layer 1 includes 1,231,072 elements and 1,294,873 nodes, while the honeycomb core includes 51,300 elements and 56,762 nodes.

**Fig 10:**
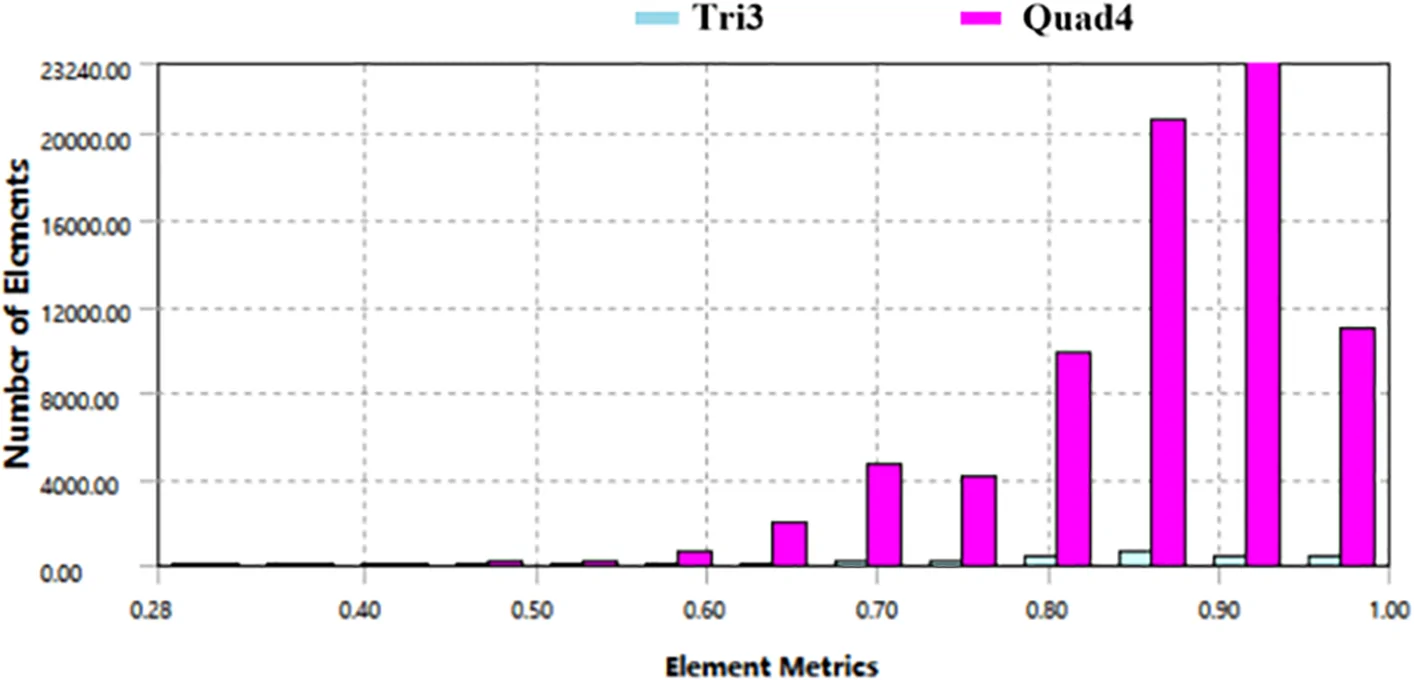
Element quality analysis.

According to the mesh quality analysis in ANSYS WorkBench, the average element quality of the finite element model is greater than 0.7, as shown in Fig 10. This indicates that the mesh division quality and quantity meet the accuracy requirements for finite element analysis.

#### 3.1.2. Natural frequency and mode shape.

To deeply understand the influence of the fiber layup sequence on the dynamic characteristics of the inner beam, finite element modeling of the inner beam was performed under two layup patterns (asymmetric: [0/45/-45/90/0/45/-45/90] and symmetric: [0/45/-45/90/90/-45/45/0]).

Low-order natural frequencies were obtained through free modal analysis.. The free mode analysis modeling in Workbench is based on the assumption of linear elastic small deformation, and the assembly adopts MPC binding contact. No constraints or loads are applied throughout the entire process, fully releasing the 6 rigid degrees of freedom of the inner beam. As shown in Table 3, the first to fifth natural frequencies of the symmetric layup are slightly higher than those of the asymmetric layup. For instance, the first natural frequency of the symmetric layup (161.08 Hz) is 4.77 Hz higher than that of the asymmetric layup (156.31 Hz), and the frequency difference between them increases with the increase in modal order. Therefore, when the inner beam uses a symmetric layup, its dynamic characteristics are superior to those of an asymmetric layup.

**Table 3:** Natural frequencies in different layup sequences Hz.

Layup sequence	1	2	3	4	5
Asymmetric	156.31	293.95	427.72	800.21	829.15
Symmetry	161.08	299.14	440.78	814.17	854.47

**Fig 11:**
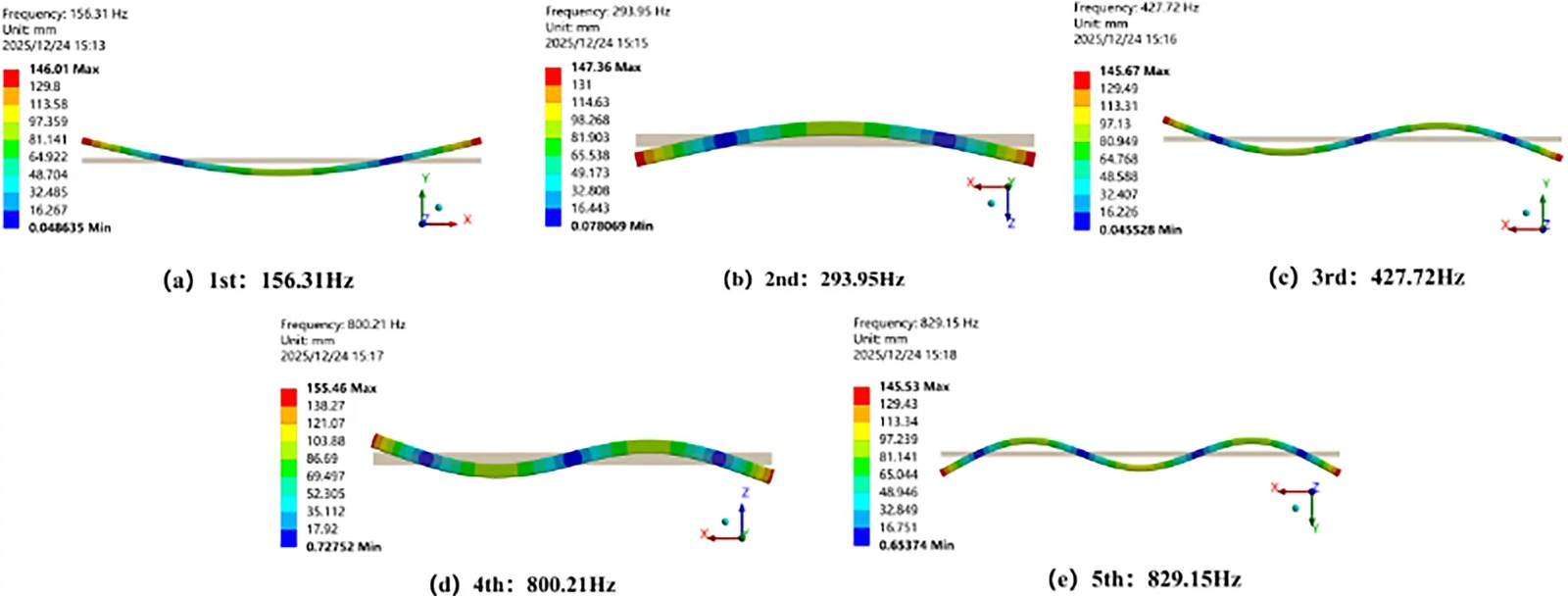
Asymmetric layup mode shape.

**Fig 12:**
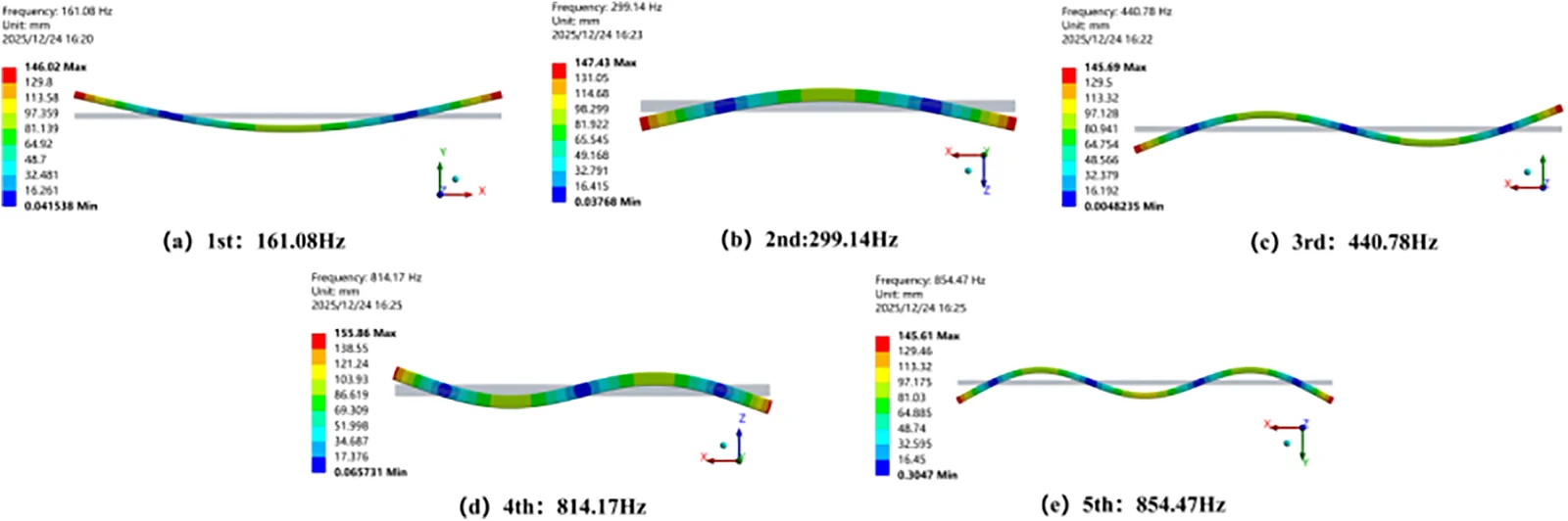
Symmetric layup mode shape.

Figs 11 and 12 show the vibration modes of the inner beam under two layup configurations. Comparison reveals that for the first to fifth natural frequencies, the asymmetric layup and symmetric layup exhibit similar vibration patterns, with maximum vibration deformation occurring at both ends of the crossbeam. The maximum deformation of the 1st to 5th mode shape ranges from 145 mm to 156 mm.

From the perspective of vibration deformation, the 1st to 5th mode shapes of the inner beam all exhibit bending with different shapes and amplitudes. Among these, the bending deformations of the first, third, and fifth orders occur in the *XY* plane, while those of the second and fourth orders occur in the *XZ* plane. Overall, under both asymmetric layup:[0/45/-45/90/0/45/-45/90](see Fig 11) and symmetric layup: [0/45/-45/90/90/-45/45/0](see Fig 12), there is no significant difference in the vibration shape of the inner beam.

### 3.2. Optimize design

#### 3.2.1. Mathematical model.

When preparing fiber-reinforced composite materials, parameters such as fiber laying thickness, laying direction, and the number of integration points of each prepreg layer significantly impact the mechanical performance of the laminated structure [16].

For the laminated structure, the in-plane mechanical behavior of each individual prepreg ply follows the orthotropic elastic constitutive law under plane stress condition. The corresponding compliance form of the constitutive equation is presented in Equation (1) [17]:


$$\begin{array}{c} \left\{\begin{array}{c} @c@\varepsilon_{x}\\ \varepsilon_{y}\\ \gamma_{xy} \end{array}\right\}=\left[\begin{array}{ccc} @ccc@1/E_{x} & -\nu_{yx}/E_{y} & 0\\ -\nu_{xy}/E_{x} & 1/E_{y} & 0\\ 0 & 0 & 1/G_{xy} \end{array}\right]\left\{\begin{array}{c} @c@\sigma_{x}\\ \sigma_{y}\\ \tau_{xy} \end{array}\right\} \end{array}$$ (1)

where:

*ε*_*x*_, *ε*_*y*_: Normal strains in the x and y material directions, respectively;

*γ*_*xy*_: Engineering shear strain in the x-y plane;

*σ*_*x*_, *σ*_*y*_: Normal stresses in the x and y material directions, respectively;

*τ*_*xy*_: Shear stress in the x-y plane;

*E*_*x*_, *E*_*y*_: Elastic moduli in the x and y material directions, respectively;

*v*_*xy*_, *v*_*yx*_: Poisson’s ratios, where *ν*_*xy*_ denotes the transverse strain in the y direction induced by a unit normal stress in the x direction, and vice versa for *ν*_*yx*_;

*G*_*xy*_: In-plane shear modulus of the orthotropic ply.

Based on the above constitutive relationship, the fiber layup parameters, including the ply layup angles, and the single-ply thickness, significantly affect the stiffness distribution, as well as the stress and strain responses of the composite laminates. Consequently, for the composite heald frame structure, its dynamic characteristics, particularly the natural frequencies, are inherently dependent on these layup parameters.

Laminated beams of composite materials are anisotropic elastic structures, and their free vibration natural characteristics are determined by the structural stiffness matrix and mass matrix. The general expression for natural circular frequency is:


$$\begin{array}{c} \omega =\sqrt{\frac{K}{M}} \end{array}$$ (2)

In Equation (2): *ω* is the natural circular frequency, *K* is the overall stiffness of the structure, and *M* is the overall mass of the structure.

Specifically, the *i*-th natural circular frequency of the structure can be formulated as an implicit function of the layup parameters, as shown in Equation (3):


$$\begin{array}{c} \omega_{i}\text{=}f\left(\alpha ,\theta \text{,}t\right) \end{array}$$ (3)

Here, the subscript *i* denotes the order of the natural mode of the heald frame.

To enhance the dynamic performance of the composite heald frame, this paper establishes a multivariate optimization model to maximize the fundamental frequency of the structure, thereby avoiding resonance risks.

According to the engineering optimization standard paradigm, a complete dynamic optimization model is constructed from three aspects: design variables, constraints, and objective functions. Select the core layer parameters that affect the dynamic performance of the crossbeam as design variables, and limit the range of values based on process requirements, as shown in Equation (4).


$$\begin{array}{c} X={\left[\alpha ,\theta ,t\right]}^{T} \end{array}$$ (4)

Constraints: 25° ≤ *α* ≤ 65°, 25° ≤ ∣*θ*∣ ≤ 65°, 0.1 mm ≤ *t* ≤ 0.3 mm.

The optimization objective is to maximize the first-order natural frequency. According to the vibration relationship *ω*_1_ = 2*πf*_1_, maximizing *f*_1_ is equivalent to maximizing the first-order natural circular frequency *ω*_1_. Therefore, the optimization objective function can be defined as shown in Equation (5).


$$\begin{array}{c} \text{max}F\left(X\right)=\omega_{\text{1}}\left(\alpha ,\theta ,t\right) \end{array}$$ (5)

Combining design variables, boundary constraints, and objective function, the standard optimization mathematical model is shown in Equation (6).


$$\begin{array}{c} \left\{ \begin{array}{c} @l@\text{max}\omega \text{\textbackslash{}hspace\{0.5em}{\}}_{\text{1}}\left(\alpha ,\theta ,t\right)\text{=2}\pi f_{\text{1}}\left(\alpha ,\theta ,t\right)\\ \begin{array}{c} \text{\textbackslash{}hspace\{1em}\;\}25^{o}\leq \alpha \leq 65^{o}\\ \text{s.t.}25^{o}\leq \left|\theta \right|\leq 65^{o} \end{array}\\ \text{\textbackslash{}hspace\{1em}\;\}0.1mm\leq t\leq 0.3mm \end{array}\right. \end{array}$$ (6)

where:

*ω*_1_: The first-order natural circular frequency (rad/s);

*f*_1_: The first order natural frequency (Hz).

The constraints come from the practical engineering limitations of carbon fiber prepreg layup process, structural forming, and mechanical properties.

Through this optimization model, we aim to adjust the fiber layup parameters to maximize the fundamental frequency of the heald frame, so as to significantly improve its dynamic characteristics. This mathematical model provides an important theoretical foundation for the design, fabrication, and dynamic optimization of the composite heald frame.

#### 3.2.2. APDL parametric modeling.

**Fig 13:**
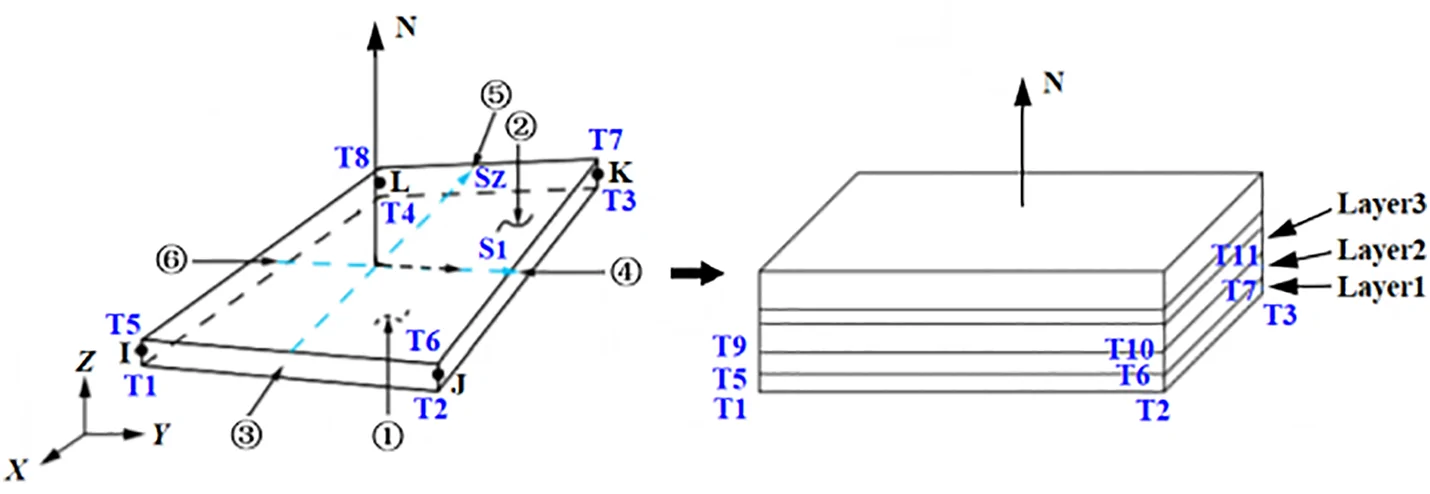
Element Shell181.

A parametric finite element modeling program based on the APDL command stream in ANSYS software is developed. The carbon fiber prepreg layup shape is simulated using the Shell181 shell element, as shown in Fig 13. The Shell181 shell element has 4 nodes (I, J, K, L), with each node possessing 6 degrees of freedom (U*X*, U*Y*, U*Z*, ROT*X*, ROT*Y*, ROT*Z*) [18]. It supports linear and nonlinear large deformation analysis and is suitable for simulating composite laminated shells or sandwich structures with thin to medium thickness.

**Fig 14:**
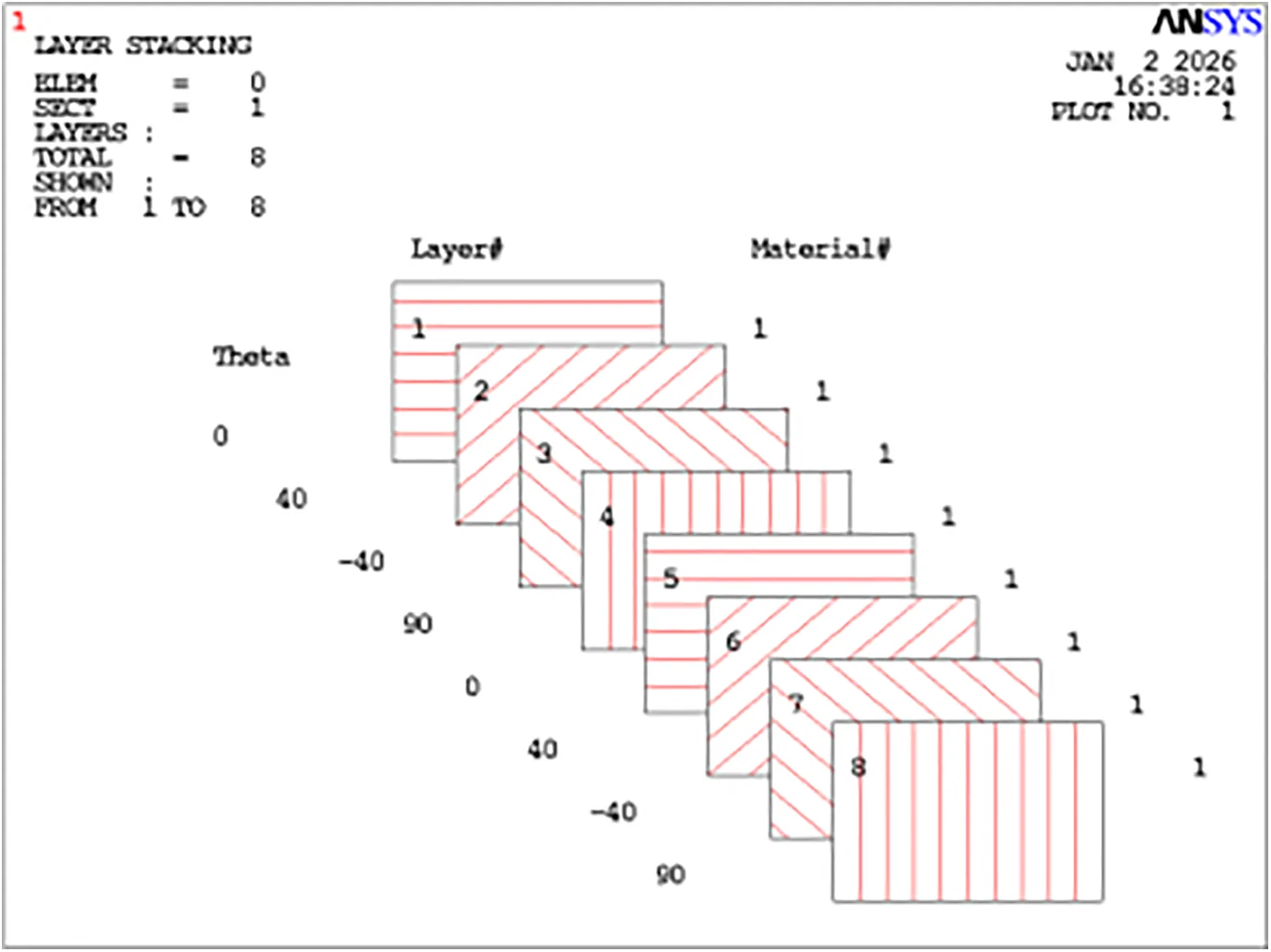
Asymmetric layup (1 ~ 8 layers).

As illustrated in Fig 14, assuming the fiber layer 1 wrapped around the outer surface of the honeycomb core adopts an asymmetric layup, with the initial layup thickness of the single carbon fiber prepreg *t* = 0.2 mm, and the initial laying angles being *α* = 40° and *θ* = −40°, the layup sequence of the carbon fiber prepregs from layer 1 to layer 8 is [0/40/-40/90/0/40/-40/90].

**Fig 15:**
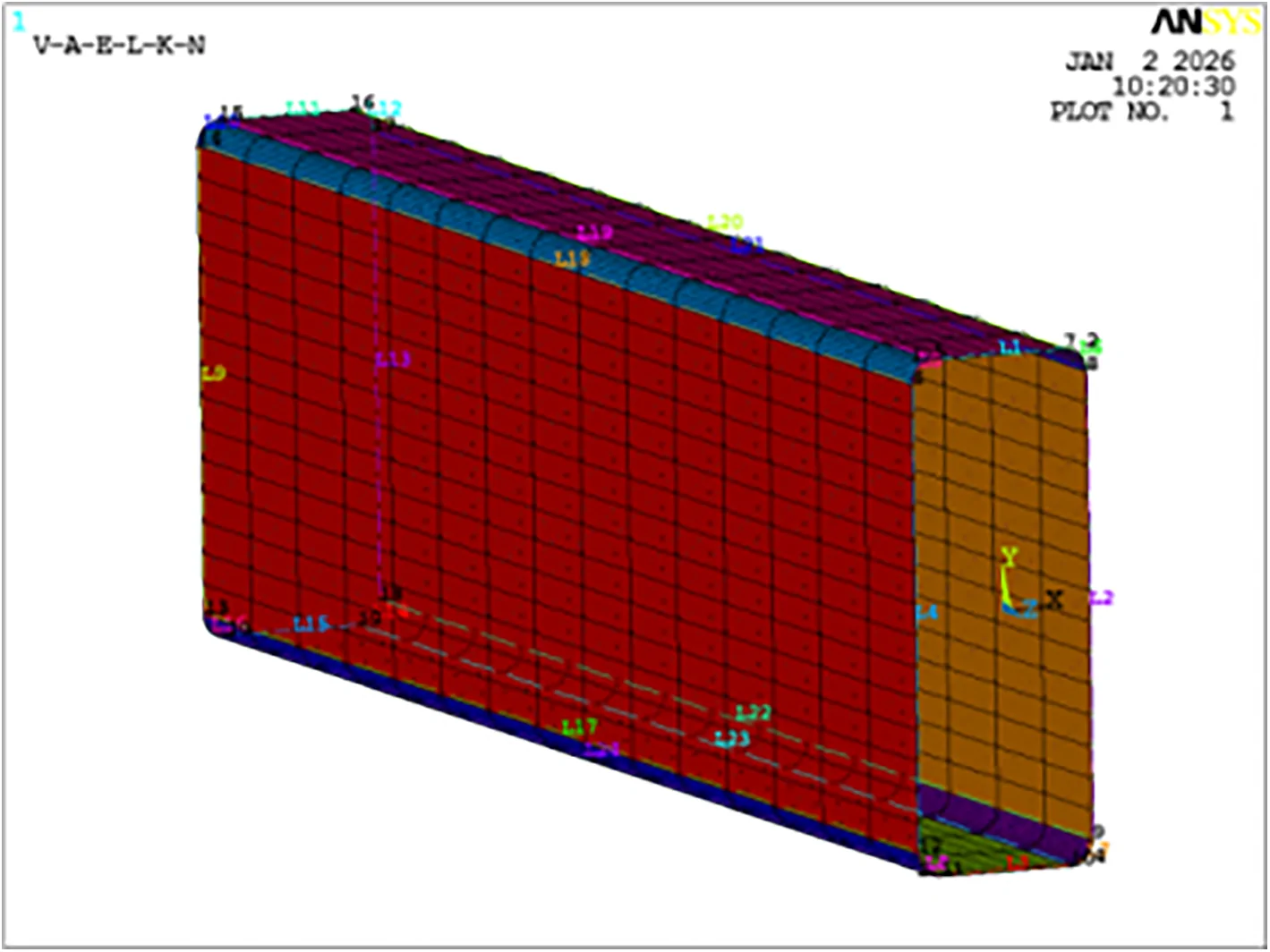
Parametric finite element model.

Fig 15 presents the parametric finite element model of fiber layer 1 (layers 1–8), established via the APDL command stream. In this model, the layup parameters of carbon fiber plies, including the fiber orientation angles (*α*, *θ*) and the single-ply thickness (*t*), are defined as design variables. The optimization objective is set to maximize the first-order natural frequency (*f*_1_).

The model in Fig 15 is discretized using Shell181 elements, with the material property assigned to Epoxy Carbon UD (230 GPa Prepreg), as detailed in Table 2. The overall thickness along the *X*-direction is 1.6 mm (corresponding to 8 layers of 0.2 mm each), and the length along the *Z*-direction is 750 mm.

#### 3.2.3. Optimize calculation.

**Fig 16:**
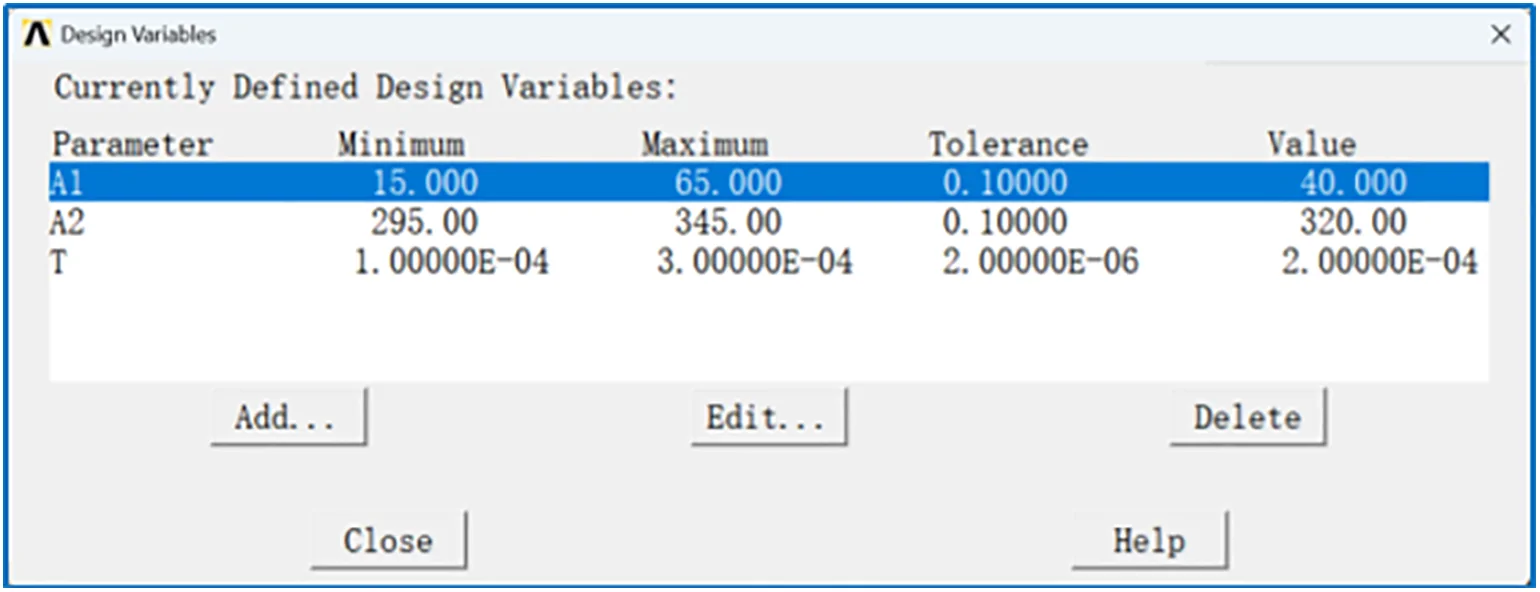
Define design variables.

Based on the initial values of *α*, *θ*, and *t*, the design variable space and state variable space were reasonably set up in the ANSYS software, as shown in Fig 16. Among them, A1 represents *α* with a range of [15°, 65°], A2 represents *θ* with a range of [295°, 345°], and T represents *t* with a range of [0.1, 0.3]mm. Through free modal analysis, the initial value of the first-order natural frequency(*f*_1_) of fiber layer 1 (layers 1 ~ 8) was obtained as 2752.2 Hz. According to this value, the goal of the optimization design is to make the parameter(*f*_1_) greater than 2752.2 Hz.

**Fig 17:**
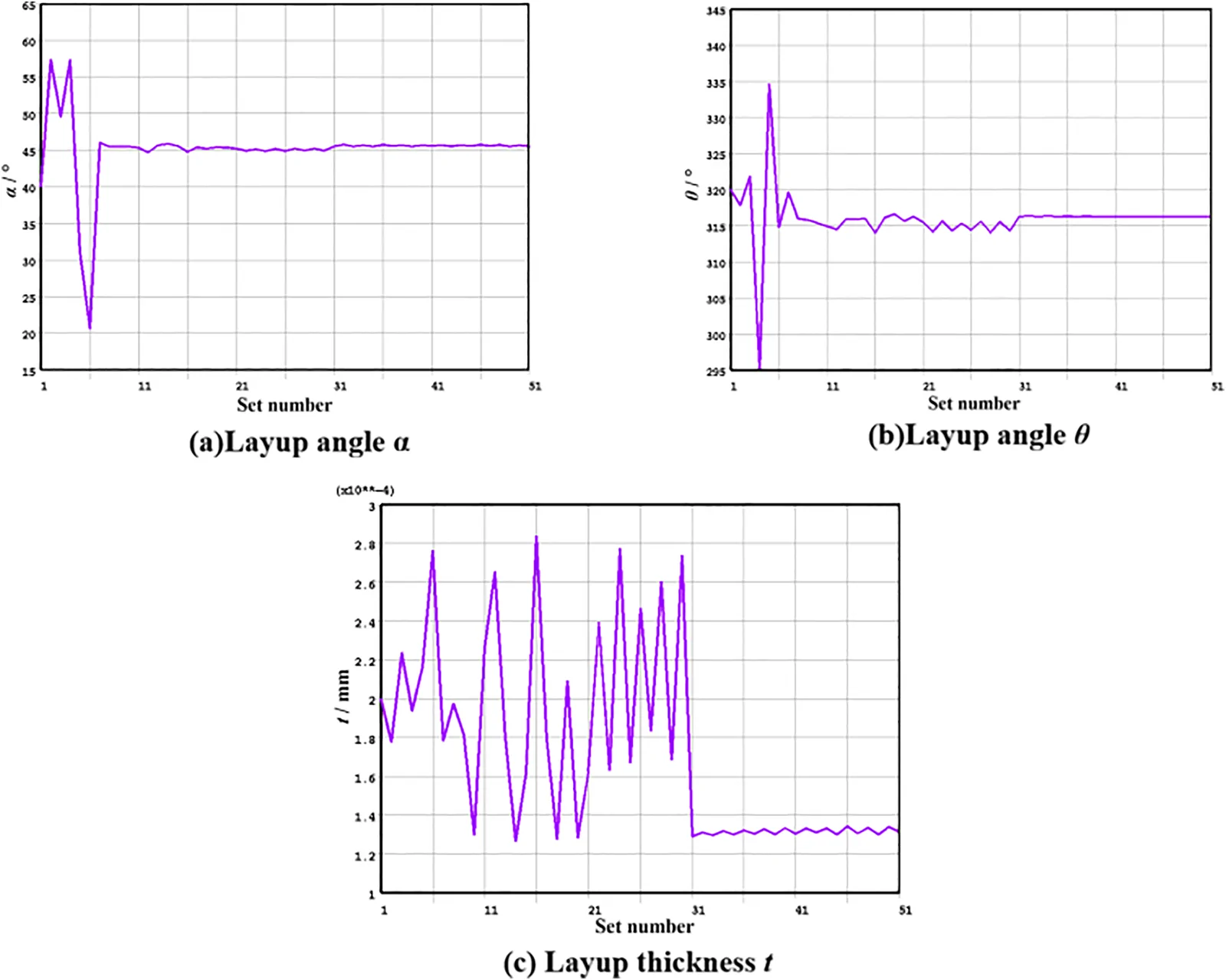
Optimization and calculation process curve. (a) Layup angle *α*, (b) Layup angle *θ*, (c) Layup thickness *t*.

Due to the presence of numerical noise and strong nonlinearity in dynamic finite element responses, the gradient information tends to be unreliable. Accordingly, it is more reasonable to employ the subproblem method, which is a gradient-free sequential approximate optimization approach featuring high robustness and efficient convergence. Using the subproblem method with the APDL command stream as shown in Fig 17, it can be clearly observed that ANSYS performed a total of 51 optimization iterations. Among them, after the 31st optimization run, the iteration curves of the design variables (*α*, *θ*, *t*) gradually stabilize, indicating that the optimal design results have achieved convergence.

#### 3.2.4. Result analysis.

**Fig 18:**
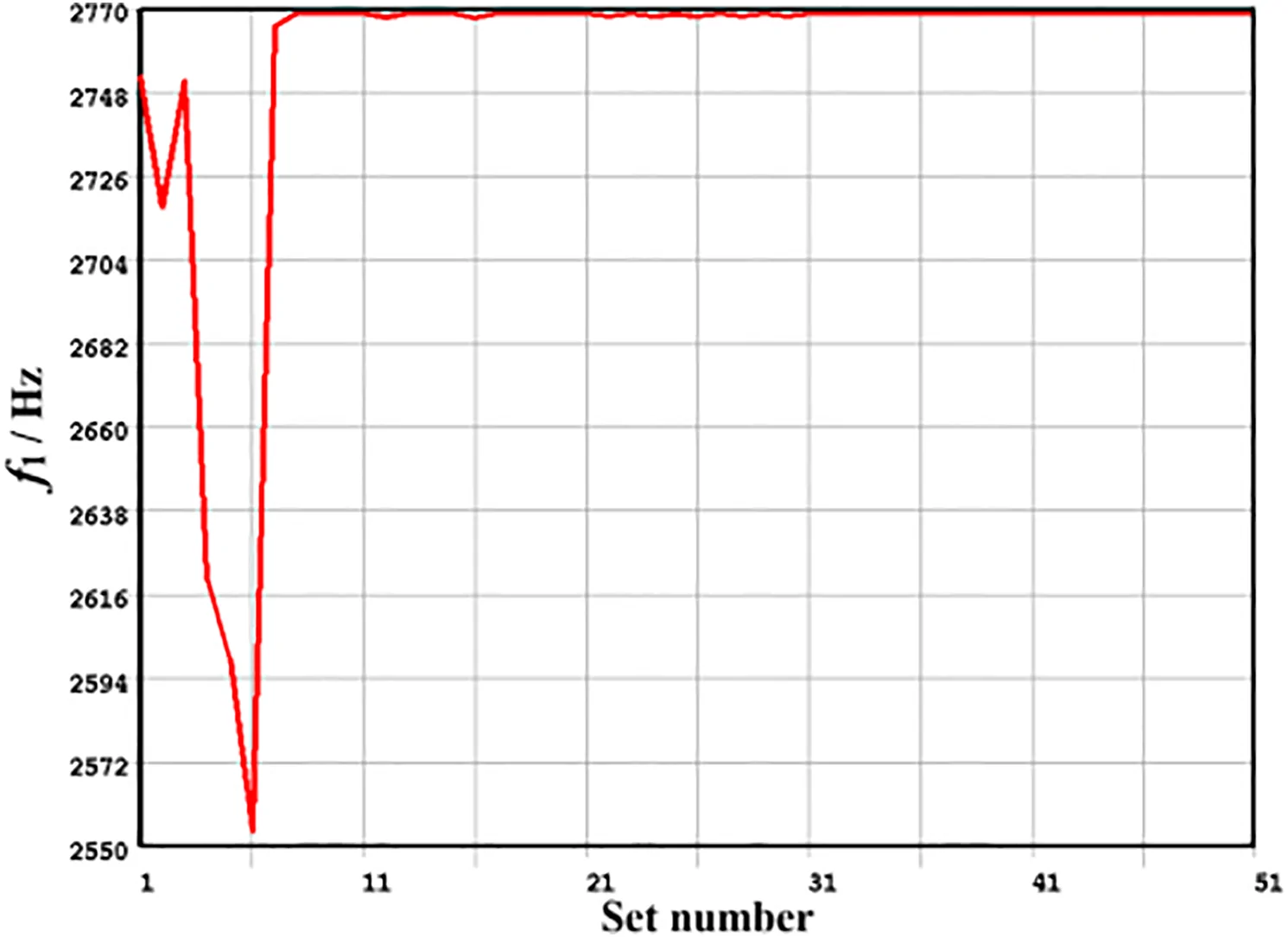
Optimize design objective *f*_1_.

As illustrated in Fig 18, after the 31st optimization iteration, the process curve of the optimized objective function (*f*_1_) tends to stabilize, eventually converging to 2768.8 Hz. Compared with the initial value (2752.2 Hz), it has increased by 16.6 Hz, which to a certain extent enhances the vibration resistance of fiber layer 1 and meets the expected optimization design requirements.

From the data in Table 4, it can be observed that when the first-order natural frequency(*f*_1_) of fiber layer 1 increases from 2752.2 Hz to 2768.8 Hz, the layup angles *α* and *θ* increase by 13.73% and 9.3%, respectively, while the layup thickness(*t*) decreases by 35%. This indicates that the fiber layup parameters(*α*, *θ*, *t*) indeed influence the increase or decrease of the parameter *f*_1_, which demonstrates the theoretical correctness and practical feasibility of the optimization mathematical model. Although the optimization results only increased the first-order natural frequency of the inner beam components by 0.6%, this can increase the first-order critical speed of the loom by about 996r/min, greatly expanding the safe operating range of high-speed looms and helping to improve weaving efficiency and stability.

**Table 4:** Optimal data of the fiber layer 1.

Variable	Initial value	Optimized value	Change rate	Change trend
*α*/^o^	40	45.49	13.73%	↑
*θ*/^o^	−40	−43.72	9.30%	↑
*t*/mm	0.20	0.13	35%	↓
*f*_1_/Hz	2752.20	2768.80	0.60%	↑

In this work, by tailoring the key layup parameters, including the single-ply thickness and layup angles of the prepreg, the dynamic performance improvement and structural weight reduction of the composite heald frame are achieved, thereby addressing the speed matching challenge between the heald frame component and the high-speed loom system.

It should be emphasized that, except for the first-order natural frequency(*f*_1_), the 2nd to 6th natural frequencies of fiber layer 1 all show an increasing trend, as shown in Table 5. Compared with the initial natural frequencies, the optimized 2nd to 6th natural frequencies increase by 40.7 Hz, 54.1 Hz, 79.2 Hz, 107 Hz, and 335 Hz, respectively. It can be seen that the increase in natural frequency is proportional to the modal order, meaning that higher modal orders correspond to greater increases in natural frequency.

**Table 5:** Natural frequencies variation Hz.

Oder	1	2	3	4	5	6
Initial value	2752.2	5854.3	7296.3	9814.6	10480	16861
Optimized value	2768.8	5895	7360.4	9893.8	10587	17196

**Fig 19:**
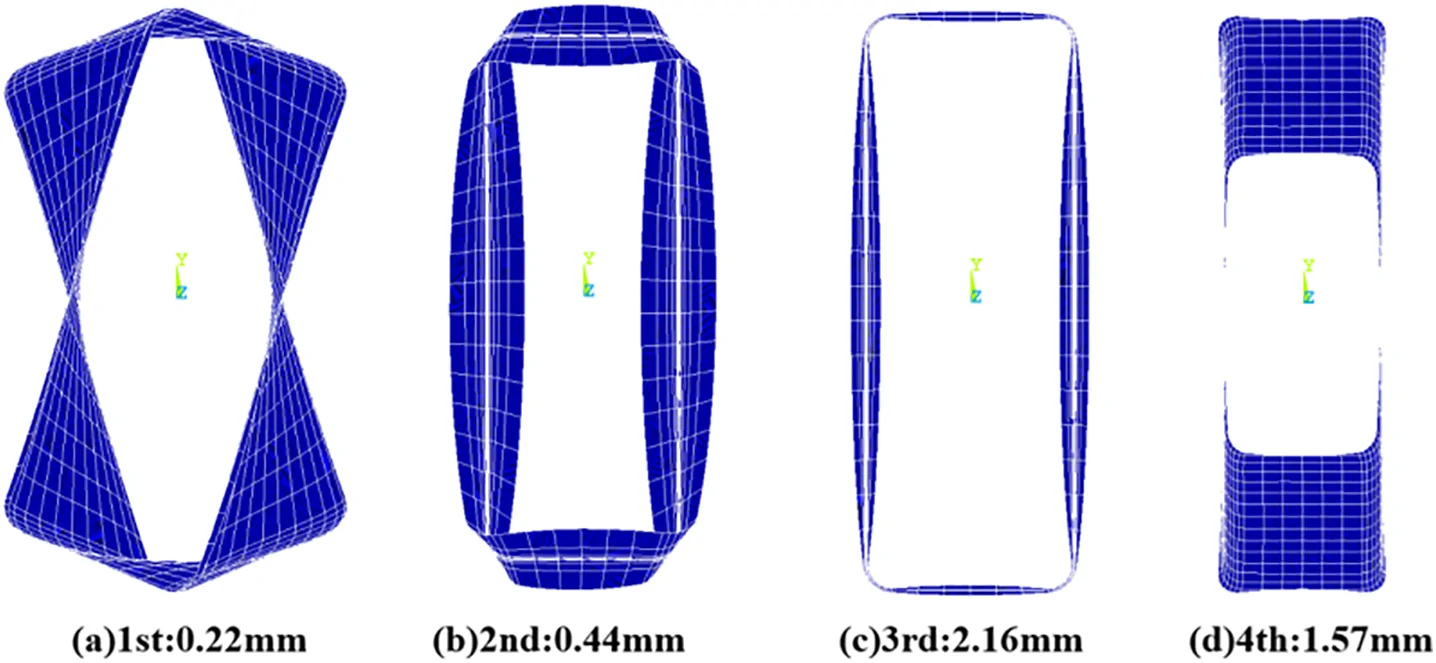
Optimized mode shapes(1st ~ 4th). (a) 1st:0.22 mm (b) 2nd:0.44 mm (c) 3rd:2.16 mm (d) 4th:1.57 mm.

Although the optimized design caused a significant change in the natural frequency of fiber layer 1, its vibration modes before and after optimization are very similar, as shown in Fig 19. It can be clearly seen that the 1st to 4th vibration modes of the optimized fiber layer 1 mainly exhibit bending and torsional deformations with different amplitudes, with the maximum deformation ranging from 0.22 mm to 1.57 mm. The initial vibration modes of fiber layer 1 (1st to 4th) are similar to those in Fig 19 and will not be elaborated further.

### 3.3. Energy consumption and efficiency

**Fig 20:**
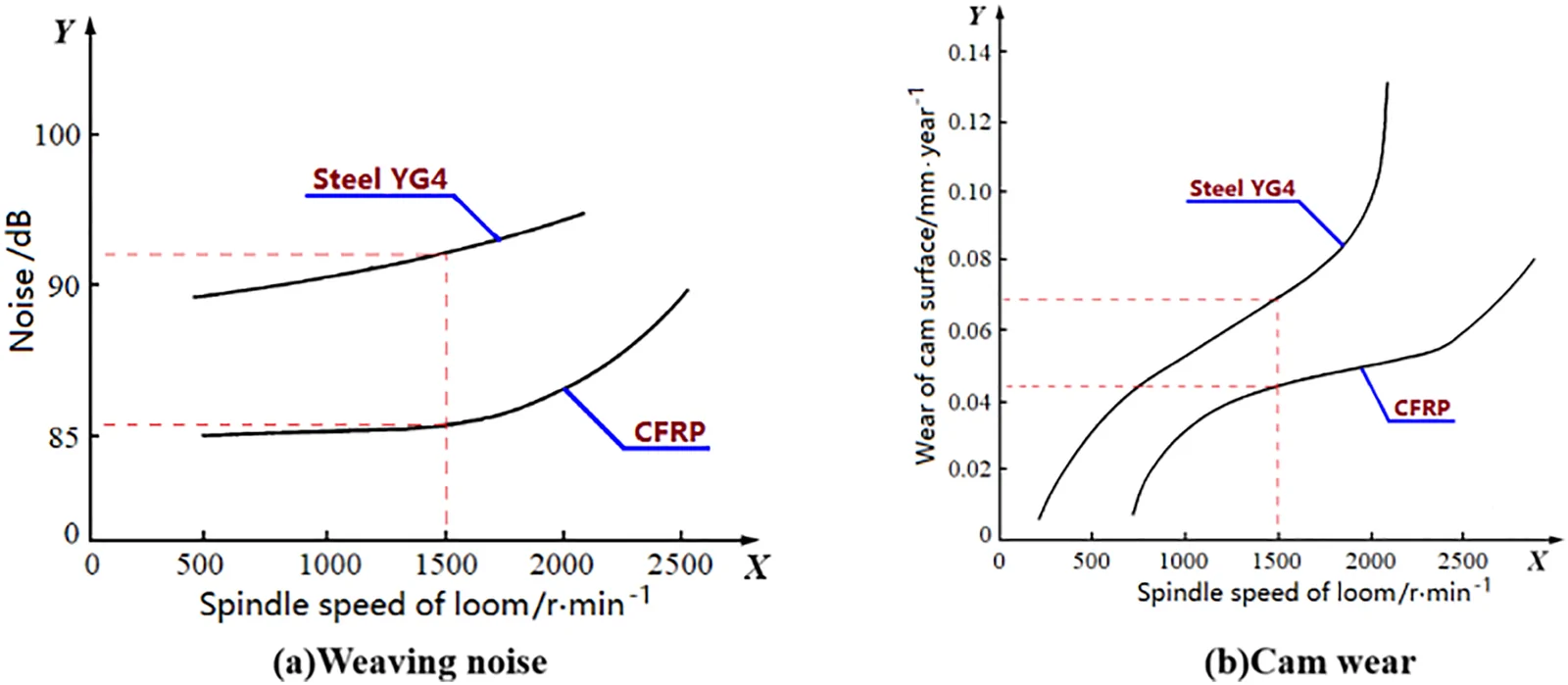
Vibration noise and wear test. (a) Weaving noise, (b) Cam wear.

Under the same loom speed, the vibration noise and component wear induced by the composite heald frame are significantly lower than those of the traditional heald frame, as illustrated in Fig 20 [4]. When the operating speed is 1500 r/min, the vibration noise of the loom system is approximately 85.4 dB (for CFRP heald frame) and 92 dB (for Steel YG4 heald frame), respectively. Meanwhile, the wear on the cam contact surface is approximately 42 μm (for CFRP heald frame) and 68 μm (for Steel YG4 heald frame), respectively.

From Fig 20(a) and (b), it can be seen that the heald frame(CFRP) not only significantly reduces the inertial load and vibration noise of the shedding system, but also provides notable advantages in energy saving, consumption reduction, and wear reduction for the loom, which conductive to improve the production efficiency and economic benefits of textile enterprises.

It should be explicitly noted that the measured performance data of the conventional YG4 steel heald frame reported in Reference [4] are adopted as the benchmark in this paper, which intuitively demonstrates the prominent advantages of the proposed carbon fiber composite heald frame in vibration and noise reduction as well as energy conservation of the loom system.

### 3.4. Preparation process

In this paper, we propose an RTM process and its implementation method suitable for preparing composite material frames, but this is only a principle introduction and does not involve specific process parameters and manufacturing steps. To achieve efficient batch manufacturing of composite heald frames, carbon fiber prepreg for the crossbeam is prepared using RTM (Resin Transfer Molding) technology. RTM is a process in which resin is injected into a closed mold to impregnate reinforcing materials, followed by curing to form the desired shape. It offers several advantages, including fast molding speed, low production costs, reduced environmental pollution, and strong adaptability to automation [19].

**Fig 21:**
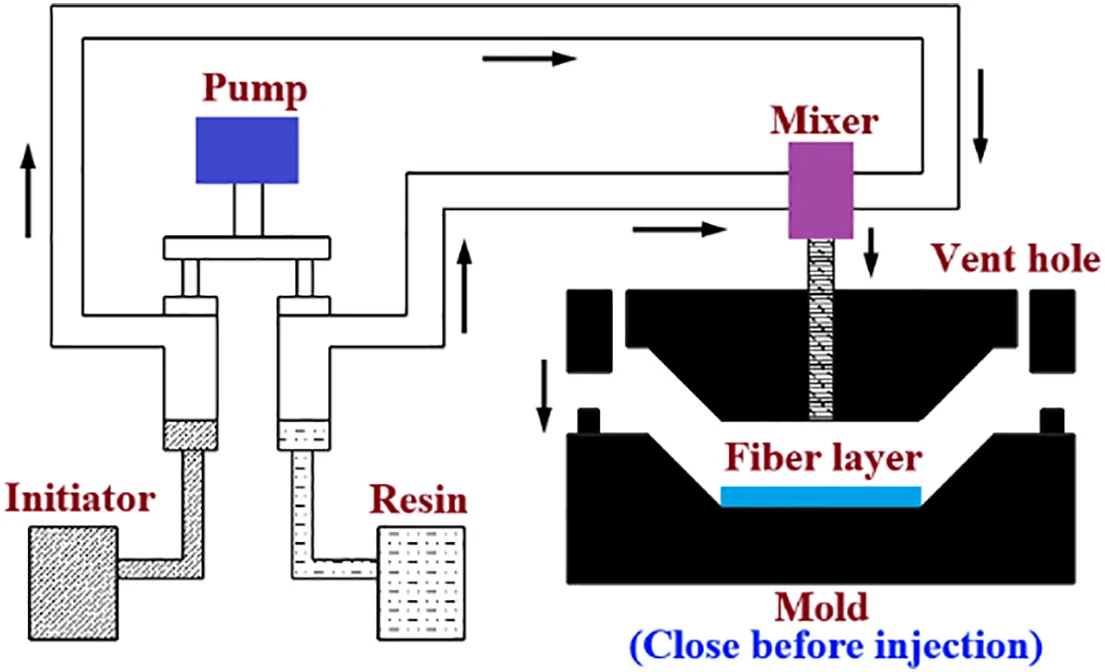
RTM process diagram.

The RTM process can integrate various fiber-reinforced materials and resin systems. Its molding principle is illustrated in Fig 21: first, the fiber-reinforced materials are laid in the mold cavity according to the preform shape. Under pump pressure or vacuum assistance, low-viscosity resin and initiator are thoroughly mixed. The mixture is then injected into the mold to expel gas from the cavity. After the resin, initiator, and fibers are fully impregnated and mixed, heating is conducted, and finally, the mixture is cured into a finished fiber-reinforced composite component.

The crossbeam is a typical planar component, and its shape meets the forming characteristics requirements of laminated structures such as panels, beams, and ribs for the RTM process. Based on the heald frame width, prepregs with different fiber layup sequences and layup thicknesses can be quickly prepared through the RTM process. On this basis, efficient automated manufacturing of sandwich delamination composite crossbeams can be achieved, thereby providing technical support for the batch production of composite heald frames.

## 4. Conclusion

This paper proposes a carbon fiber composite heald frame with sandwich delamination. In-depth research is conducted on the design methods and performance advantages of this heald frame from aspects of structural principles, layup methods, mathematical models, dynamic optimization, economy, and batch preparation processes, providing strong support for the technological innovation and practical application of composite heald frames. The research conclusions are as follows:

(1) Through finite element modeling and dynamic analysis, it was found that the dynamic characteristics of the symmetric layup [0/45/-45/90/90/-45/45/0] are superior to those of the asymmetric layup [0/45/-45/90/0/45/-45/90]. The inner beam exhibits similar vibration modes (1st to 5th orders) under both layup configurations, and all demonstrate bending deformation with varying amplitudes.
(2) Based on the layup principle and preparation process of carbon fiber prepreg, a mathematical model applicable to the dynamic optimization of composite crossbeam is proposed. A parameterized finite element model for the laminated structure (fiber layer 1) is developed using the APDL command stream. The theoretical correctness and implementation feasibility of this mathematical model are verified through an optimization design example.
(3) Optimization design results indicate that by controlling the prepreg fiber layup parameters (*α*, *θ*, *t*), the dynamic characteristics of the composite crossbeam can be effectively enhanced. The natural frequency increment of fiber layer 1 is proportional to its modal order, meaning that higher modal orders correspond to greater natural frequency increments.
(4) Compared with traditional heald frames, composite heald frames with sandwich delamination exhibit numerous advantages, including high strength, low vibration, and low noise. Additionally, they can reduce the weight of heald frames of the same specification (e.g., aluminum alloy heald frames) by approximately 25%, which contributes to vibration reduction, noise reduction, energy conservation, and reduced wear of the loom system.
(5) A preparation process for laminated structures used in composite crossbeams, known as Resin Transfer Molding(RTM), has been developed in this work, providing technical reference for the efficient batch manufacturing of composite heald frames.

## 5. Future work

Production practice has shown that applying carbon fiber composite materials to the design and manufacturing of heald frames can achieve structural lightweighting, while substantially reducing inertial loads and vibration noise during the shedding motion. Undoubtedly, composite heald frame plays an important positive role in improving the working environment of the weaving workshop and the physical and mental well-being of workers, which is also the practical significance of this research work.

Nevertheless, due to limitations such as research funding, material costs, and experimental conditions, this paper still has certain deficiencies in the experimental study of composite heald frames. In the future, our team will integrate actual weaving production, build upon existing research, concentrate resources and efforts to trial-produce full-scale composite heald frame test specimens, and continue in-depth research and exploration in this field.

## Supporting information

S1 File Heald frame. (RAR)
